# Supplementary material for: The interactome of intact mitochondria by cross-linking mass spectrometry provides evidence for coexisting respiratory supercomplexes
Source: Mol Cell Proteomics. 2017 Dec 8;17(2):216–32. doi: 10.1074/mcp.RA117.000470 (PMC5795388; doi:10.1074/mcp.RA117.000470)
Supplement: Supplemental Data [file supp_RA117.000470_133922_0_supp_23978_fzffwf.zip › spectra_annotation/mito_spectra_annotation/475-1-4-1-7-1.pdf]

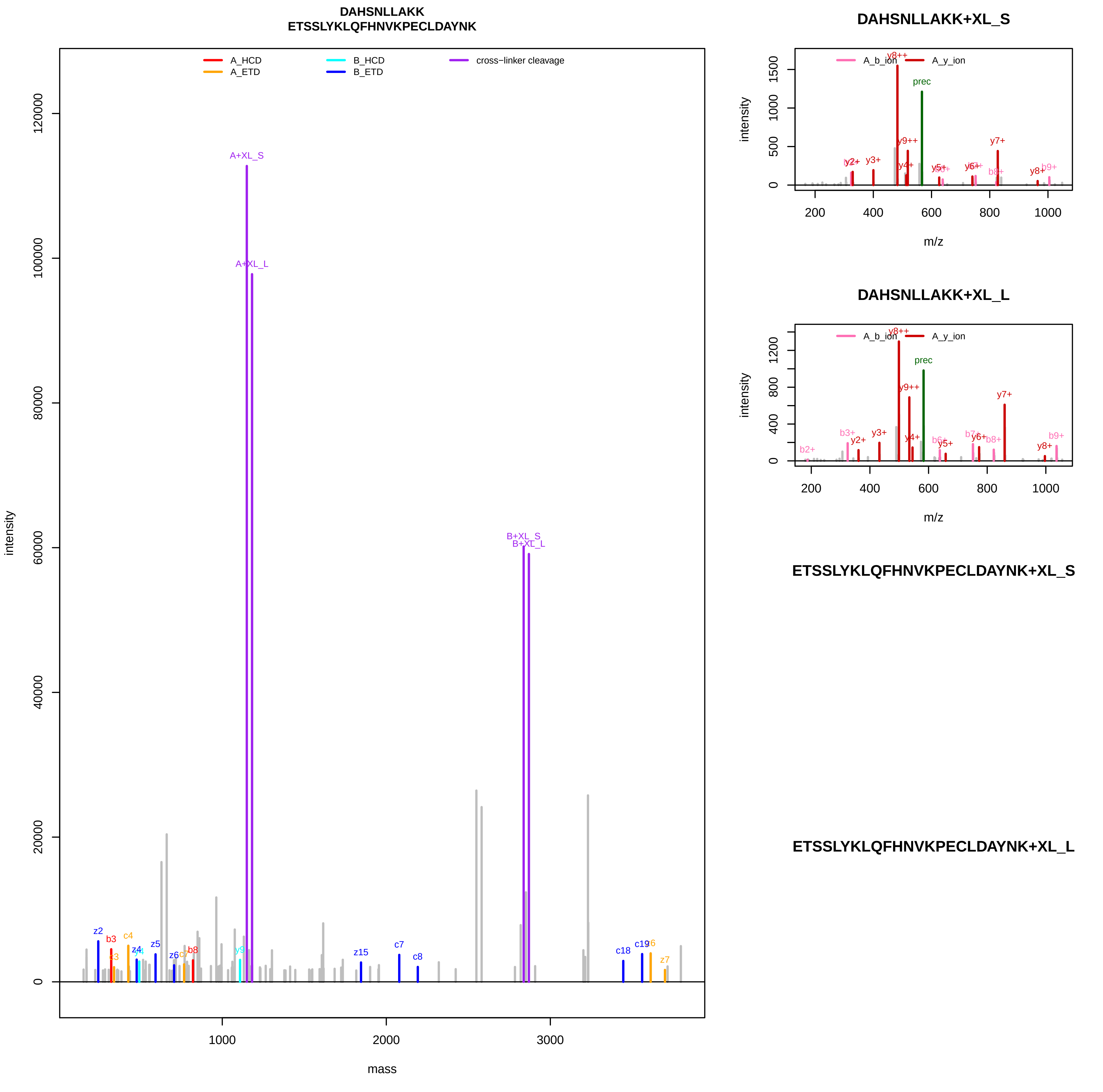
DAHSNLLAKK
ETSSLYKLQFHNVKPECLDAYNK
A_HCD
A_ETD
B_HCD
B_ETD
cross−linker cleavage
A+XL_S
A+XL_L
B+XL_S
B+XL_L
z2
b3
c3
c4
z4
z5
z6
b8
y9
z15
c7
c8
c18
c19
z7
intensity
mass
0
20000
40000
60000
80000
100000
120000
1000
2000
3000
DAHSNLLAKK+XL_S
A_b_ion
A_y_ion
y8++
prec
y9++
y2+
y3+
y4+
y5+
y6+
y7+
b8+
y8+
b9+
intensity
m/z
0
500
1000
1500
200
400
600
800
1000
DAHSNLLAKK+XL_L
A_b_ion
A_y_ion
y8++
prec
y9++
b2+
b3+
y2+
y3+
y4+
b6+
y5+
b7+
y6+
b8+
y7+
y8+
b9+
intensity
m/z
0
400
800
1200
200
400
600
800
1000
ETSSLYKLQFHNVKPECLDAYNK+XL_S
ETSSLYKLQFHNVKPECLDAYNK+XL_L
